# Prevalence of anxiety, depression and post-traumatic stress disorder in the Kashmir Valley

**DOI:** 10.1136/bmjgh-2017-000419

**Published:** 2017-10-15

**Authors:** Tambri Housen, Annick Lenglet, Cono Ariti, Showkat Shah, Helal Shah, Shabnum Ara, Kerri Viney, Simon Janes, Giovanni Pintaldi

**Affiliations:** 1 Medical Department, Medecins Sans Frontieres, New Delhi, India; 2 National Centre for Epidemiology and Population Health, Australian National University, Canberra, Australian Capital Territory, Australia; 3 Public Health Department, Artsen zonder Grenzen, Amsterdam, Netherlands; 4 Medical Statistics, London School of Hygiene and Tropical Medicine, London, UK; 5 Department of Psychology, University of Kashmir, Srinagar, India; 6 N/A, Medecins Sans Frontieres, Srinagar, India

**Keywords:** health services research, public health, cross-sectional survey

## Abstract

**Background:**

Following the partition of India in 1947, the Kashmir Valley has been subject to continual political insecurity and ongoing conflict, the region remains highly militarised. We conducted a representative cross-sectional population-based survey of adults to estimate the prevalence and predictors of anxiety, depression and post-traumatic stress disorder (PTSD) in the 10 districts of the Kashmir Valley.

**Methods:**

Between October and December 2015, we interviewed 5519 out of 5600 invited participants, ≥18 years of age, randomly sampled using a probability proportional to size cluster sampling design. We estimated the prevalence of a probable psychological disorder using the Hopkins Symptom Checklist (HSCL-25) and the Harvard Trauma Questionnaire (HTQ-16). Both screening instruments had been culturally adapted and translated. Data were weighted to account for the sampling design and multivariate logistic regression analysis was conducted to identify risk factors for developing symptoms of psychological distress.

**Findings:**

The estimated prevalence of mental distress in adults in the Kashmir Valley was 45% (95% CI 42.6 to 47.0). We identified 41% (95% CI 39.2 to 43.4) of adults with probable depression, 26% (95% CI 23.8 to 27.5) with probable anxiety and 19% (95% CI 17.5 to 21.2) with probable PTSD. The three disorders were associated with the following characteristics: being female, over 55 years of age, having had no formal education, living in a rural area and being widowed/divorced or separated. A dose–response association was found between the number of traumatic events experienced or witnessed and all three mental disorders.

**Interpretation:**

The implementation of mental health awareness programmes, interventions aimed at high risk groups and addressing trauma-related symptoms from all causes are needed in the Kashmir Valley.

**Key questions**

**What is already known about this topic?**

- Populations living in protracted conflict are at higher risk of developing signs and symptoms of mental illness.
- There is a recognised need for research that describes the epidemiology of mental illness in a population in order to direct provision of mental health services and inform policy.
- The few studies conducted on this topic in the Kashmir Valley lack generalisability and comparability, limited by the use of non-validated screening tools, small samples sizes or non-probability based sampling. 

**What are the new findings?**

- Our study is the first representative study estimating the prevalence of mental distress in all 10 districts of the Kashmir Valley, using culturally adapted, translated and validated screening tools.
- Our findings show that adults living in all 10 districts of the Kashmir Valley experience high levels of anxiety, depression and PTSD symptoms. The cumulative impact of multiple traumatic events (natural and manmade) was shown to be a significant predictor of mental distress.

**Recommendations for policy**

- Our findings suggest that an increase in coverage and access to mental health services should be prioritised in settings that have been affected by natural and man-made disasters, together with minimising the repeated exposure to traumatic events.
- Interventions should be focused on decentralising access to mental health services and community mental health promotion activities to strengthen individual and community coping strategies.
- Those identified as at increased risk for mental distress should be targeted when formulating policy and planning culturally sensitive interventions.

## Introduction

Globally, psychological disorders make up a large proportion of disease burden and are recognised as the leading cause of years of life lived with a disability (Disability-Adjusted Life Years).^1 2^ This results in decreased productivity and has a negative impact on the quality of life of affected individuals and their families.^1 2^ In recognition of mental health as a priority public health problem, in 2012 the World Health Assembly called for a comprehensive, coordinated response from health and social sectors to address mental health disorders at the country level.^3^ In order to target services and inform policy decisions, the epidemiology of psychological distress in a given population must be understood. Furthermore, in countries and contexts affected by conflict, the combination of exposure to traumatic experiences, restrictions on economic development and the breakdown of traditional social support mechanisms place the population at increased risk of psychological distress.^4–6^

Following the partition of India in 1947, the Kashmir Valley has been subject to continual political insecurity and ongoing conflict.^7^ In 1989, an insurgency began leading to the displacement of over 100 000 Kashmiri Pandits and 27 years of militant and military activity.^8 9^ By 2015, approximately 70 000 Kashmiris had lost their lives in the conflict and 8000 people had been reported missing.^10^ The effect of prolonged exposure to violence on the psychological well-being of the population has been confounded by natural disasters such as a 7.6 Mw magnitude earthquake in 2005 and floods in 2014, in addition to livelihood factors such as poverty and unemployment.^11–15^

Increasing evidence on the impact of protracted conflict and natural disasters on the mental health of the Kashmiri population is available. The Institute of Mental Health and Neurosciences (IMHANS) in the valley’s major city, Srinagar, has observed a rise in outpatient presentations for mental health issues from an average of 100 per week in 1980 to between 200 and 300 per day in 2013.^16^ In addition, the number of suicide attempts increased by more than 250% between 1994 and 2012^17^ and other studies report a high prevalence of traumatic experiences and associated symptoms of mental illness.^17 18^ Prior studies conducted in the Kashmir Valley estimating the prevalence of mental distress have been limited by non-probability based sampling, small sample sizes and the use of non-validated instruments to measure mental distress have hampered the generalisability of the results.^12 19 20^

Therefore, we aimed to estimate the prevalence of psychological distress (specifically symptoms of anxiety, depression and PTSD) across all 10 districts of the Kashmir Valley in order to assist mental health service providers to increase the relevance and impact of current activities in Kashmir and to advocate for early and targeted interventions and evidence-based policies.

## Methods

### Study design and participants

This population-based cross-sectional survey was conducted between October and December 2015 in the 10 districts of the Kashmir Valley. We adopted a multistage random sampling design.^21 22^ The sampling frame for each of the 10 districts was obtained from the 2011 census data with *villages* (in rural areas) and *wards* (in urban areas) as enumeration areas.^23^ In the first stage of sampling, we drew a random sample of 40 villages/wards per district using probability sampling proportional to size. In the second stage of sampling, we used two methods; in rural areas we drew a random sample of 14 households per village based on the list of households kept by the village head. In urban areas, we randomly generated 14 global positioning system points per randomly selected ward and the household closest to the point was chosen for interview. In the final stage of sampling, one member of the household, 18 years of age or older, was randomly selected for interview using a random numbers table.

A household was defined as a group of persons who slept under the same roof more for than three months in the past 12 months and who shared the same cooking pot. We aimed for 560 participants per district, based on a sample size calculation which assumed a precision estimate of ±6%, a 95% CI, an estimated pooled prevalence of mental distress of 40%^20 24 25^ and the number of interviews the team could feasibly conduct during the time period they were present in the village. This yielded a total sample size of 5600 individuals. The sampling of each district separately allowed for the estimation of separate district-level prevalence rates, in addition to pooled prevalence rates. Due to resource limitations and travel distances, it was not possible to return to the household on a subsequent day and so in the event that no one in the household was home on the day of interview or if the household did not want to participate a replacement household was selected. This was the household on the immediate right of the non-participating household.

The study was approved by the Médecins Sans Frontières Ethics Review Board (ERB) (ID 1516), the Government Medical College Srinagar ERB (ID 19/ETH/GMC/ICMR) and the Australian National University Human Research Ethics Committee (ID 2015/516).

### Procedures

Two electronic questionnaires were administered in face-to-face interviews during the survey: the Household Demographics Questionnaire (HDQ) and the Personal Interview Questionnaire (PIQ). The questionnaires were converted into an electronic version using Open Data Kit (ODK) software. The questionnaires were uploaded onto tablets and data were collected by entering responses directly into the tablet. These questionnaires were developed by the study team using an iterative process involving multiple methods of free-listing, focus group discussions and review by an expert panel.

The HDQ, administered to the self-identified head of the household, included information on demographic characteristics, family history of psychological illness and the household’s dependence on other persons for living. The PIQ was administered to the randomly selected individual ≥18 years of age, in the same household. Individuals were asked questions on the following topics: additional demographic information, ability to function in daily life, self-reported physical health, problems of daily life, coping strategies and exposure to traumatic events. In addition, the Hopkins Symptom Checklist (HSCL-25) for anxiety and depression, and the Harvard Trauma Questionnaire-16 (HTQ-16) for post-traumatic stress disorder (PTSD) were administered. These instruments were culturally adapted and translated; this process has been described elsewhere.^26^

The daily functionality checklist was created during pre-survey free-listing interviews and focus group discussions (these results are not presented here). The traumatic events checklist was adapted from the Life Events Checklist (LEC). The LEC traumatic events includes natural disasters, conflict-related trauma, traumatic life experiences such as accidents and life-threatening illness or injury, sexual trauma and death. Prior to the survey, a technical working group extended the section on conflict-related trauma to include specific traumatic experiences relevant to the Kashmir Valley context, including crackdowns, frisking, interrogation with threats to life, torture, disappearance of friends or family, loss of property or belongings, forced separation from family members and direct combat exposure such as militant or military attacks. Respondents reported on one of four categories per event; (1) personally experienced this event, (2) witnessed this event happening to someone else, (3) know of someone this happened to and (4) don’t know anyone this has happened to. The number of exposures over the respondent’s lifetime was reported.

The HSCL-25^27^ is composed of 10 items designed to assess symptoms of anxiety and 15 items assessing symptoms of depression in the prior four weeks. Rating is via a four-point Likert scale with categories of response being: ‘never or no’, ‘sometimes’, ‘often’, or ‘always’. Three scores are calculated from the responses: the depression score (the average of the 15 depression items), the anxiety score (the average of the 10 anxiety items) and the total score (the average of all 25 items). Across various population groups, the total score has been shown to be highly correlated with severe emotional distress of an unspecified diagnosis.^27^ The anxiety items are consistent with the Diagnostic and Statistical Manual IV (DSM-IV) diagnosis of generalised anxiety disorder; however, the symptoms may also be consistent with other anxiety disorders.^27^ The depression score is correlated with major depressive disorder as defined by the DSM-IV.^27^ The HTQ-16,^27 28^ is the fourth section of a larger instrument which addresses 30 trauma symptoms derived from the DSM-IV criteria for PTSD. It is often used in isolation as a screening instrument for symptoms of PTSD. The checklist is composed of 16 items rated on a four-point Likert scale, similar to the HSCL-25. The DSM-IV PTSD score is calculated from averaging the scores, with a higher score suggesting an increased probability of PTSD.^27^

In recognition of the importance of culturally validating instruments, we conducted a separate study where employment of rigorous methodological approaches in the cultural adaptation, translation and validation of the HSCL-25 and HTQ-16 followed four phases taken from sequences recommended by Brislin (1976), Van Ommeren (1999) and Flaherty *et al*. 29 This methodology is explained elsewhere.^26^

Briefly, from a sample of 304 individuals, the Kashmiri optimal cut-point for maximising the sensitivity and specificity of the HSCL-25 anxiety subscale was 1.75 (95% CI 1.64 to 1.86) and the depression subscale 1.57 (95%CI 1.47 to 1.67). The conventional cut-point for both scales is 1.75.^27^ A Kashmiri cut-point was not estimated for the HTQ-16 due to too few observations classified by the gold standard psychiatric interview as a case for PTSD, we adopted the recommended international cut-point of 2.0.^27^ The receiver operating curve analysis demonstrated good diagnostic accuracy, with the area under the curve 0.81 and 0.82 for the anxiety and depression subscale, respectively. Cronbach’s alpha of the HSCL-25 was calculated to be 0.92 and Cronbach’s alpha for the HTQ-16 was calculated to be 0.90, demonstrating high internal reliability. The HSCL-25 and the HTQ-16 are not diagnostic tools; diagnosis can only be confirmed via clinical interview with a psychologist/psychiatrist. Therefore, the term ‘probable case’ is used throughout, for persons scoring above the screening instrument cut point, with the recognition that use of screening tools also captures individuals with sub-syndromal illness. 30

Enumerators fluent in Kashmiri, Urdu and English were recruited from a pool of postgraduate sociology, social work and psychology students form Kashmir University. A total of 53 enumerators were trained to administer the questionnaires using the tablets. A pilot survey was conducted on 1st and 2nd of October 2016 in 54 households in one Ward of Srinagar; these results were not included in the survey sample. Based on the results of the pilot study, no revisions to the questionnaire were deemed necessary. Data collection was carried out by 10 teams, each consisting of one team leader and four enumerators with an equal male to female ratio. Interviews were conducted in Kashmiri, Urdu or English and strict privacy was ensured. Completed versions of the questionnaire were uploaded daily by team leaders from the tablets to a secure server based at the Medecins Sans Frontieres (MSF) office in New Delhi, India. Data were password protected and only accessed by the principal investigator.

A research information sheet was provided to prospective participants in Urdu and English (Kashmiri is the most widely spoken language, whereas Urdu and English are the prominent languages of literacy). Due to low literacy rates, participants were read the information sheet prior to being asked to sign or mark a translated consent form. No incentive was offered for participation in the survey. In the event a respondent become distressed during the interview, the interview was stopped and psychological first aid was administered by the trained enumerator. The interview was only continued if the respondent was willing and able to continue answering the questions. A mental healthcare referral service was integrated into the survey design by way of a referral card with contact details for all mental health service providers in the valley. Participants showing distress were advised to contact the nearest mental health service provider and directed to the MSF 24-hour mental health hotline. An MSF clinical psychologist was on-call throughout the survey to provide telephone support to survey enumerators when required.

### Statistical analysis

Observations from participants who refused to respond to at least one item on either the HSCL-25 or HTQ-16 were excluded from the analysis. Data analysis was conducted in Stata V.13.1 (StataCorp, Texas, USA).^31^ We conducted descriptive analysis on respondent characteristics using frequencies, means, SD and 95% CI. Data were then weighted to account for the sampling design and for the over-representation of females in the sample, and prevalence estimates were calculated for probable anxiety, depression and PTSD using the prior stated cut-points. We used the complex survey design command in Stata to apply probability weights. Sensitivity tests were conducted on sample weights to assess differences in prevalence estimates after poststratification weighting on gender. SEs were estimated using the Taylor series linearisation method to adjust for design effects.^32^ The Wilcoxon-Mann-Whitney test was utilised to compare continuous variables. The Χ^2^ 2was used to test for associations between categorical variables. The psychometric properties of the instruments were evaluated using Cronbach’s alpha for internal reliability, where an alpha of 0.7 was considered to be an acceptable reliability coefficient and 0.9 or larger, excellent reliability. 33

Univariate logistic analysis was used to calculate crude ORs with 95% CIs, identifying evidence of association with study outcomes (depression, anxiety and PTSD) and a priori risk factors, that is, sex, age group, marital status, education, main daily activity, rural or urban residence and exposure to traumatic events. Variables that showed a significant association with the outcome of interest, with a p value of less than 0.25 were included in the multivariable analysis; with separate models created for each study outcome. 34–36 Adjusted ORs and adjusted Wald tests were calculated.^35^ Forward step-wise regression was utilised to build the final main effects model.^35^ Backward step-wise regression was then used to remove variables with an adjusted Wald test > 0.05. In the final model, only exposure factors which had adjusted ORs >1.0 with a p*-*value <0.05 were considered statistically significant predictors of the study outcomes.^35^ Variance inflation factors were estimated to check for collinearity.^35^

## Results

**Figure 1:**
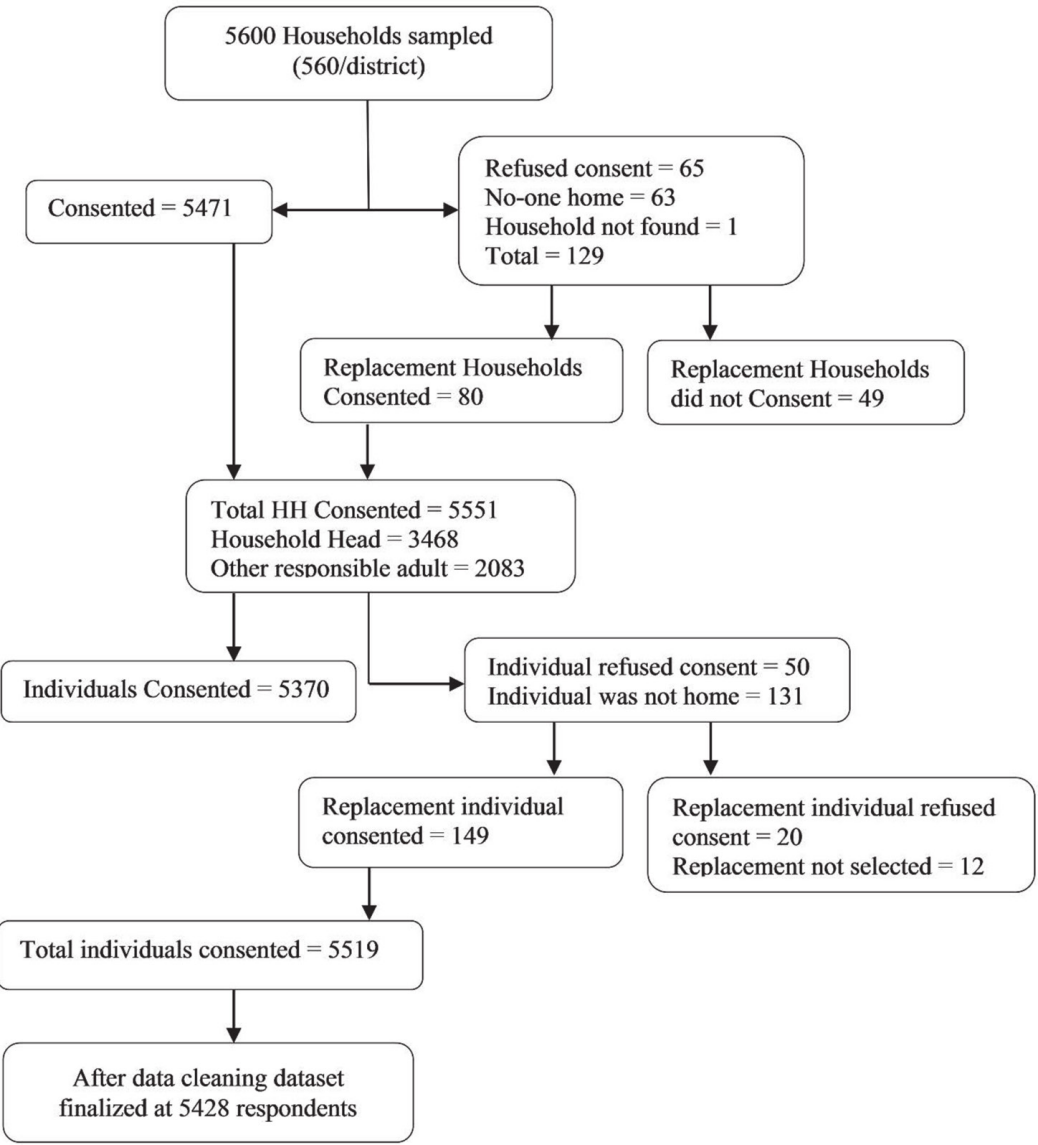
Flow diagram of response rates for the Kashmir Mental Health Survey, 2015.

We collected data from 399 villages across all ten districts of the Kashmir Valley, one village was not accessible due to heavy snowfall. Of the 5600 households selected and approached for interview, 65 randomly selected households and 49 replacement households refused consent, providing an overall household participation rate of 97.9% (5551 households). Of the 5551 individuals invited to participate, 70 did not consent for interview, providing an individual participation rate of 98.7%. Nine interviews were discontinued due to respondent distress and 54 participants refused to respond to at least one item on either the HSCL-25 or HTQ-16 and thus were excluded from the analysis. After data cleaning, a sample of 5428 observations was available for analysis (figure 1).

The average household size was 6.5 persons and 27% (n=1466) of households reported that at least one person in their family had suffered from a psychological illness. Most households reported being self-sufficient (81%, n=4398) with 95% (n=5171) stating that the family always has at least two meals per day. A higher proportion of respondents lived in a rural area (78%, n=4216).

The mean age of respondents for the individual interview was 38 years (SD 15.4) with similar age distributions in men and women (table 1). The majority of respondents were married (68.4%, n=3706) and 65% (n=3509) of all respondents were women. Men reported higher educational attainment than women, with a high proportion of women reporting no formal education (41.8%, n= 1460). Some form of employment (full time, contract work or self-employed) or working in a family business was reported by 60% (n=1154) of male respondents, while the majority of women (81.0%, n=2843) reported home duties as their main activity.

**Table 1:** Demographic characteristics of respondents, Kashmir Mental Health Survey, 2015

	Total (N=5428)	Males (n=1919)	Females (n=3509)
	Proportion n (%)	Proportion n (%)	Proportion n (%)
Mean age (SD)	38.2 (15.4)	40.6 (17.1)	37.0 (14.2)
Age group			
18–34 years	2449 (45.1)	814 (42.4)	1635 (46.6)
35–54 years	1943 (35.8)	617 (32.2)	1326 (37.8)
55+ years	1036 (19.1)	488 (25.4)	548 (15.6)
Main activity			
Some employment	886 (16.3)	725 (37.8)	161 (4.6)
Student	705 (13.0)	320 (16.7)	385 (11.0)
Family business	449 (8.3)	429 (22.4)	20 (0.6)
Unemployed/retired	385 (7.1)	285 (14.9)	100 (2.8)
Home duties	55∙3 (55.3)	157 (8.2)	2843 (81.0)
Marital Status			
Never married	1438 (26.5)	579 (30.2)	859 (24.5)
Married	3706 (68.4)	1275 (66.5)	2431 (69.4)
Widowed/divorced/separated	275 (5.1)	62 (3.2)	213 (6.1)
Education			
None	1899 (35.1)	439 (22.9)	1460 (41.8)
Primary	775 (14.3)	322 (16.8)	453 (13.0)
High	1694 (31.4)	715 (37.4)	979 (28.1)
Graduate	787 (14.6)	390 (20.4)	397 (11.4)
Vocational	248 (4.6)	48 (2.5)	200 (5.7)
Area of residence			
Rural	4216 (77.7)	1535 (80.0)	2681 (76.4)
Urban	1212 (22.3)	384 (20.0)	828 (23.6)
District of residence			
Kupwara	524 (9.7)	190 (9.9)	334 (9.5)
Kulgam	535 (9.9)	180 (9.4)	355 (10.1)
Ganderbal	538 (9.9)	201 (10.5)	337 (9.6)
Badgam	538 (9.9)	201 (10.5)	337 (9.6)
Baramulla	543 (10.0)	185 (9.6)	358 (10.2)
Shopiyan	543 (10.0)	176 (9.2)	367 (10.5)
Anantnag	550 (10.1)	197 (10.3)	353 (10.1)
Pulwama	550 (10.1)	194 (10.1)	356 (10.1)
Bandipora	553 (10.2)	221 (11.5)	332 (9.5)
Srinagar	554 (10.2)	174 (9.1)	380 (10∙8)

The majority (99.2%) of the adult study population experienced or witnessed at least one traumatic event during their lifetime (range 1–19), with an average of 7.7 (SD 4.0) traumatic events per person. Men reported that they had witnessed or experienced more traumatic events than women (mean 8.4 (SD 4.2) and 6.4 (SD 3.8), respectively; p<0.001) (table 2). In table 3, the weighted lifetime prevalence of traumatic events experienced and witnessed by respondents are summarized; 70.6% of adults recounted having experienced or witnessed the sudden or violent death of someone they knew and a further 75.7% reported having experienced a work-related accident, transport accident and/or a life-threatening illness or injury.

**Table 2:** Weighted proportion of traumatic events experienced or witnessed by respondents in the Kashmir Mental Health Survey, by sex, 2015

	Total (N=5428)		Men (n=1919)		Women (n=3509)		Difference (%)	Overall p-Value
	%	SE	%	SE	%	SE		
Traumatic events								<0.001
No traumatic events	7.5	0.002	5.6	0.002	11.1	0.003	5.5	
1–2 traumatic events	9.7	0.006	7.3	0.008	14.7	0.010	7.4	
3–5 traumatic events	23.5	0.009	19.4	0.012	31.8	0.010	12.4	
6–10 traumatic events	39.7	0.010	41.0	0.014	37.1	0.011	3.9	
>10 traumatic events	26.3	0.010	31.7	0.014	15.4	0.010	16.3	

The poststratified gender weighted mean score on the HSCL-25 was 1.55 (SD 0.47, 95% CI 1.53 to 1.57). Female respondents scored higher than male respondents (females: 1.65, 95% CI 1.62 to 1.67; males: 1.50, 95% CI 1.47 to 1.53, p<0.001). The weighted mean score on the HSCL-25 Anxiety items (items 1–10) was 1.50 (SD 0.48, 95% CI 1.48 to 1.52). Female respondents scored higher than male respondents (females: 1.63, SD 0.50, 95% CI 1.61 to 1.66; males: 1.44, SD 0.43, 95% CI 1.41 to 1.46, p<0.01). Cronbach’s alpha for the items on the anxiety subscale was 0.90. The weighted mean score for the HSCL-25 Depression items (items 11–25) was 1.58 (SD 0.51, 95% CI 1.56 to 1.60). Female respondents scored higher than male respondents (females: 1.66, SD 0.51, 95% CI 1.63 to 1.68; males: 1.54, SD 0.50, 95% CI 1.51 to 1.57, p<0.01). Cronbach’s alpha for the items in the depression subscale was 0.87. The mean score for the HTQ-16 was 1.55 (SD 0.41, 95% CI 1.52 to 1.57). Female respondents scored higher than male respondents (females: 1.61, SD 0.50, 95% CI 1.58 to 1.63; males: 1.55, SD 0.47, 95% CI 1.48 to 1.55, p<0.01). Cronbach’s alpha for the HTQ-16 was estimated at 0.89, demonstrating good internal reliability.

The weighted population prevalence rate for psychological distress (ie, for symptoms of depression, anxiety and PTSD combined) based on the cut points of the screening instruments was 45% (95% CI 42.6 to 47.0). Forty-one per cent (95% CI 39.2 to 43.4) exhibited signs of probable depression, 26% (95% CI 17.5 to 21.2) signs of probable anxiety and 19% (95% CI 23.8 to 27.5) signs of probable PTSD. Sensitivity analyses using unweighted data and data weighted only for the sampling design yielded similar findings; however, poststratification weighting for gender yielded lower prevalence estimates (refer to Supplementary file 1).

10.1136/bmjgh-2017-000419.supp1 Supplementary file 1

Over half of survey respondents (64%) reported feeling low in energy and worrying too much (61%) in the four weeks prior to the survey. A large proportion also indicated that in the previous four weeks they had experienced difficulty sleeping (41%), a loss of interest in things (44%) and feelings of sadness (49%), worthlessness (46%) and crying easily for no identified reason (41%).

**Table 3:** Weighted lifetime prevalence of traumatic events experienced or witnessed, by sex, Kashmir Mental Health Survey, 2015

	Total (N=5428)		Men (n=1919)		Women (n=3509)		% Difference	95% CI	p-Value
	(%)	SE	(%)	SE	(%)	SE			
Natural disaster related	93.5	0.006	93.5	0.008	93.5	0.007	0.0	−1.4 to 1.5	0.927
Conflict related	93.0	0.006	94.2	0.008	90.4	0.007	3.8	2.7 to 5.5	<0.001
Crackdowns, round-up raids, frisking	81.1	0.010	82.7	0.012	77.6	0.011	5.1	2.5 to 6.7	
Fire or explosion	73.4	0.011	78.6	0.014	62.8	0.012	15.9	14.3 to 19.2	
Militant or military attacks	41.8	0.011	46.8	0.016	31.7	0.012	15.1	11.8 to 17.3	
Assault with a weapon	33.7	0.011	38.6	0.016	23.6	0.011	15.0	10.9 to 16.1	
Interrogation or harassment with threat to life	33.0	0.010	36.0	0.014	26.7	0.010	9.3	8.8 to 14.1	
Captivity, that is, kidnapped/imprisoned/ held hostage	32.4	0.011	36.7	0.015	23.5	0.011	13.2	10.4 to 15.7	
Torture	27.9	0.010	31.2	0.014	21.3	0.010	9.9	8.8 to 13.9	
Death of a loved one	70.6	0.011	74.0	0.014	63.6	0.012	10.4	7.8 to 12.9	<0.001
Sudden death	59.5	0.012	62.4	0.016	53.6	0.013	8.8	6.1 to 11.6	
Violent death	48.7	0.011	51.7	0.015	42.7	0.012	9.0	6.0- to 11.6	
Separation from loved one	25.7	0.010	28.2	0.014	20.6	0.010	7.6	5.3 to 10.2	<0.001
Disappearance	15.7	0.008	17.1	0.011	12.0	0.008	5.1	3.0 to 7.1	
Forced separation	15.4	0.009	16.9	0.011	13.3	0.008	3.6	1.7 to 6.0	
Life trauma	75.7	0.009	80.8	0.012	65.4	0.011	15.4	12.5 to 17.3	<0.001
Transport accident	54.2	0.011	63.0	0.014	36.2	0.012	26.8	23.1 to 28.6	
Life-threatening illness	45.6	0.012	48.8	0.016	39.1	0.012	9∙6	6.3 to 11.9	
Work accident	41.8	0.011	45.4	0.015	34.5	0.011	10.9	9.0 to 14.6	
Sexual trauma	11.1	0.007	12.2	0.010	9.0	0.008	3.2	1.7 to 5.3	0.007
Sexual assault	9.2	0.007	10.3	0.009	7.0	0.007	3.3	2.4 to 5.7	
Bad sexual experience	8.3	0.006	9.1	0.009	6.9	0.006	2.2	0.8 to 4.0	
Physical trauma	60.2	0.001	66.4	0.016	47.3	0.012	19.1	14.8 to 20.3	<0.001
Loss of property/belongings	51.1	0.014	53.6	0.018	45.9	0.015	7.7	5.5 to 11.2	<0.001

We found a high prevalence of mental health comorbidity among people with probable depression, anxiety and PTSD; 89% of respondents identified with probable PTSD were also identified with probable depression, and 71% were classified with probable anxiety. Similarly, 90% who met the criteria for probable anxiety also met the criteria for probable depression; 16% of respondents were identified as probable case for all three disorders. Furthermore, 12% of the Kashmiri adult population responded positively to the question from the HSCL-25, *In the past four weeks how often have you had thoughts of killing yourself*; 94% of these respondents were classified as a probable case for at least one of the three disorders.

Tables 4a–c show the adjusted ORs from multivariate analysis examining the associations with all three disorders. Associations were identified with being female, increasing age, being divorced, widowed or separated, having a lower educational attainment and living in a rural area. When assessing the association between the number of traumatic events and psychological distress, a dose–response effect was observed; as the number of traumatic events experienced increased, so did psychological distress. This was consistent across all three psychological disorders. Experiencing or witnessing greater than six traumatic events over a lifetime significantly increased the odds of anxiety, depression and PTSD (Tables 4a–c).

**Table 4a:** Weighted adjusted ORs of probable depression by demographic characteristics in survey respondents,Kashmir Mental Health Survey, 2015

Variables	OR	95% CI		p-Value
Sex				
Male	1.00			<0.001
Female	1.73	1.39	2.16	
Age group				
18–34 years	1.00			0.1221
35–54	1.11	0.92	1.34	0.2930
55+	1.31	1.01	1.69	0.041
Education				
None	1.39	1.10	1.76	<0.001
Primary	1.00			
Secondary	0.64	0.50	0.82	
Graduate	0.40	0.29	0.54	
Vocational	1.75	1.16	2.65	
Main activity				
Family business	0.67	0.48	0.94	0.006
Employed	1.00			
Household duty	1.15	0.88	1.49	
Student	1.20	0.90	1.60	
Unemployed/retired	1.35	0.97	1.87	
Traumatic events				
No trauma	0.95	0.37	2.41	<0.001
1–2 traumatic events	1.00			
3–5 traumatic events	1.79	1.35	2.38	
6–10 traumatic events	3.86	2.95	5.06	
>10 traumatic events	6.42	4.75	8.66	

**Table 4b:** Weighted adjusted ORs of probable anxiety by demographic characteristics in survey respondents, Kashmir Mental Health Survey, 2015

Variables	OR^†^	95% CI		p*-*Value
Sex				
Male	1.00			<0.001
Female	1.88	1.43	2.47	
Education				
None	1.46	1.14	1.86	<0.001
Primary	1.00			
Secondary	0.76	0.59	0.98	
Graduate	0.40	0.28	0.57	
Vocational	1.69	1.14	2.51	
Main activity				
Employed	1.00			0.033
Family business	0.70	0.48	1.01	
Student	1.17	0.82	1.67	
Unemployed/retired	1.16	0.80	1.68	
Household duty	1.31	0.94	1.84	
Area				
Rural	1.29	1.05	1.59	0.015
Urban	1.00			
Traumatic events				
No trauma	1.34	0.55	3.26	<0.001
1–2 traumatic events	1.00			
3–5 traumatic events	1.97	1.48	2.62	
6–10 traumatic events	3.56	2.71	4.66	
>10 traumatic events	6.10	4.50	8.27	

**Table 4c:** Weighted adjusted ORs of probable post-traumatic stress disorder by demographic characteristics in survey respondents, Kashmir Mental Health Survey, 2015

Variables	OR	95% CI		p*-*Value
Sex				
Male	1.00			<0.001
Female	2.01	1.65	2.45	
Age group				
18–34	1.00			0.018
35–54	1.22	0.98	1.52	
55+	1.47	1.12	1.92	
Marital status				
Not married	1.00			0.004
Married	1.18	0.92	1.52	
Widowed/separated/divorced	1.98	1.30	3.02	
Area				
Rural	1.54	1.20	1.98	0.001
Urban	1.00			
Traumatic events				
No trauma	0.43	0.05	3.47	<0.001
1–2 traumatic events	1.00			
3–5 traumatic events	2.14	1.39	3.31	
6–10 traumatic events	6.32	4.07	9.80	
> 10 traumatic events	12.39	7.89	19.44	

## Discussion

The results presented in this study support findings from other research conducted in the Kashmir Valley with the added strength of providing scientifically robust and generalisable estimates of mental distress in all 10 districts. In 2008, Yaswi and Haque^20^ concluded that a ‘high’ number of victims of war associated trauma suffer from PTSD symptoms with those reporting personal experience directly related to the conflict suffering from chronic depression. However, the non-probability sample and small sample size of 80 individuals limited the generalisability of the results. The tools used included the Beck Depression Inventory and the Everstine Trauma Response Index-Adapted, neither of which had been validated for the Kashmiri context. Other studies have used purposive sampling to target victims of conflict related trauma and assess the presence of psychiatric symptomology in this target group with non-standardised questionnaires.^12^ Khan (2013) measured mental health outcomes in 390 probability sampled urban households in four administrative regions of Srinagar.^37^ While the author does not state what tools were used in the survey he concludes that 46% of the sample suffered from anxiety and 32%, depression. Between 2003 and 2005, Margoob *et al*^38^ used clinical interviews conducted by psychiatrists to assess the prevalence of PTSD in 2391 probability sampled individuals from six districts of the Kashmir Valley. Prevalence of PTSD was found to be 7% with a life-time prevalence rate of PTSD reported at 15%.^38^ Using the Self Reporting Questionnaire (SRQ) and probability sample of 510 households in two districts in the Kashmir Valley in 2005, De Jong *et al*^24 25^ reported that psychological distress was experienced by 33% of their sample, with one-third reporting suicidal ideation. While cut-off scores were adapted from the previously validated Indian SRQ, these were not validated specifically for the Kashmiri context.^24 25^ Research has also been conducted on the impact of natural disasters on mental health in the Kashmir Valley. However, research limitations include small sample size and the lack of use of standardised and validated instruments.^39^

Our findings are consistent with those reported in population-based mental health surveys in other comparable/similar settings. Yasan *et al*^40^reported a prevalence rate of 15% for current PTSD in a population affected by protracted conflict in Turkey. In southern Sudan, Roberts *et al*^41^ reported the prevalence rates for PTSD and depression at 36% and 50%, respectively.^41^ A more recent study conducted by Ayazi estimated the prevalence rate of PTSD as 26% in the southern Sudanese population.^42^ In Afghanistan, prevalence estimates for depression range from 39% to 68%, anxiety 52%-72% and PTSD 20%-42%.^43 44^

Available mental health services in the Kashmir Valley follow a western biomedical model of care and treatment. Services are largely centralised in the main city of Srinagar. There is one dedicated psychiatric hospital, IMHANS, which provides inpatient and outpatient care. Other major hospitals in Srinagar also offer psychiatric services, with a few psychiatrists operating private clinics. Decentralised services are limited to a pool of Kashmiri psychiatrist and psychologists rostered to hold outpatient clinics at some of the district hospitals at set days of the week. The WHO has strongly advocated for the introduction of mental health in primary healthcare^45^ with research reporting successful implementation of primary care mental health programmes^46 47^; however, few primary care workers know how to recognise an individual with mental health issues. In 1999, the government of India initiated the District Mental Health Plan (DMHP) with the intention of staggering a rolling out of community-based mental health services in all states of India.^48^ The programme commenced in Jammu/Kashmir in 2004–2005, however, the 2012 National Mental Health Plan (NMHP), report results from a review of the DMHP, stating it was barely functional in most districts.^48^ The 2012 NMHP suggests a renewed commitment by the government of India to address the mental health needs of its population and calls for research which can ‘offer insights as well as pathways for change’.^48^

Our findings highlight areas to target for mental health intervention programmes. The association between psychological distress and older age, lower levels of education and being divorced, widowed or separated has also been found in studies conducted in other contexts such as Sri Lanka^49^, Yugoslavia^50^, Iraq^51^, Afghanistan^44 52^ and Turkey^40^. Policies and services directed at improving literacy and education outcomes, targeting the older generation, and increased community and social support for those having lost or separated from a marital partner may serve to improve psychological well-being and resilience. We recommend interventions start with meaningful community engagement, beginning with those most at risk and extending to the community with a holistic approach to improving mental health, moving away from the biomedical model of individualistic care and treatment.

Reduced exposure to traumatic life events could have a significant impact on the psychosocial well-being and recovery of individuals living in contexts experiencing political insecurity. Our results suggest the majority of the adult population in the Kashmir valley have experienced or witnessed multiple traumatic events during their lifetime. A dose-response relationship between the number of traumatic events experienced and the development of PTSD is also reported in the literature.^53^ The psychological impact of traumatic experiences on an individual can result in delayed manifestation of symptoms, which can take years to present, which is exacerbated by further exposure to trauma.^53^ A study conducted in Yugoslavia concluded that people with untreated conflict-related PTSD have a high risk of still having PTSD more than ten years after the traumatic event.^50^ The high prevalence of probable PTSD (19%) in the Kashmiri population may reflect the impact of cumulative exposure to traumatic events, delayed manifestation of symptoms, a longstanding disorder that has not been treated, lack of access to care and treatment or a combination of these and other factors. The cross-sectional nature of this study limits our ability to provide evidence on the long-term impact of the protracted conflict in the Kashmir Valley, a longitudinal study would be a worthy research endeavour.

Although men reported a higher number of experienced or witnessed traumatic events compared with women, the prevalence of probable PTSD was higher among women. This difference between males and females has been reported in other published studies and in other cultures.^42 51^ The increased vulnerability of women to PTSD is not well understood and requires further research.^54^ Higher prevalence rates of psychological distress among women compared with men is commonly reported in the literature.^54–56^ Consultation with Kashmiri mental health practitioners revealed the following possible explanations specific to the Kashmiri population: alexithymia (difficulty in experiencing, expressing and describing emotional responses) is more common among Kashmiri men and can lead to underreporting on questions associated with quantifying emotional responses, as men may be perceived to be ‘weak’ if they show emotions. Ventevogel *et al*^57^ proposed similar reasoning for differences in prevalence of mental distress in men and women in Afghanistan, suggesting the possibility of gender-specific interpretations of psychologically oriented questions, such as questions about ‘feeling sad’ or ‘crying often’. The differences in male and female emotional expression, and what is socially acceptable as an expression of mental distress, may have led men to underreport some symptoms, in order not to feel ashamed in front of the interviewer.^57 58^ Sociocultural factors common to Kashmiri society and others in the region could also bear impact on women’s ability to access support. Kashmiri men have more opportunities to move around outside of the home, whereas women are largely confined to domestic chores and responsibilities, having limited social interaction outside the home. Further research should examine the gendered nature of features of distress.

Silove *et al*^5^ linked psychological distress in a conflict affected population in Timor-Leste with feelings of uncertainty about the future and persistent feelings of injustice (connected to perpetrators not being prosecuted and held account for violations of human rights), feelings of vulnerability associated with concern for personal safety and the safety of loved ones. Jayasuriya *et al*^49^ linked psychological distress in Sri Lanka with the close proximity of army camps to civilian homes and a persistent perceived threat to the safety of self and loved ones as factors which exacerbate and prolong mental distress in the population. The Kashmir Valley is known as one of the most highly militarised regions of the world.^10^ The impact of living in close proximity to army camps, feelings of vulnerability and of injustice need to be explored in future research with respect to the impact on the mental health of the population.

This study had some limitations. The use of etic screening instruments to measure the prevalence of psychological distress can lead to an overestimate of the true prevalence of disease. We attempted to minimise this by conducting a separate study; in which we culturally adapted and translated the HSCL-25 and HTQ-16 for the Kashmiri population prior to conducting the survey.^26^ The advantage of using these instruments was that they have been widely used cross-culturally in a variety of contexts affected by protracted conflict^28 57 59–66^, enabling us to compare our findings with those of other surveys. Trauma, is a third variable with a known and well-established impact on mental health in conflict affected populations. 6 67 68 The strong relationship demonstrated in this study with trauma and mental distress, adds strength to the external validity of the HSCL-25 and HTQ-16 as a measure of mental distress in the Kashmiri population. The cross-sectional nature of our study prevents conclusions on causes of psychological distress; we are only able to report associations. Due to restrictions imposed by travel times and security, survey teams could not re-visit selected enumeration areas. While every effort was made to locate the selected individual for interview, the substitution of unavailable randomly selected individuals for another randomly selected individual may have led to the over-representation of women in our sample. We used poststratification weights on gender to correct for this in analysis; however, we acknowledge that interviewing individuals only available at the day and time we visited the village may have impacted on the representativeness of our sample.

The main purpose of this study was to provide baseline epidemiological data on mental distress in the Kashmiri Valley. The study has highlighted a high level of psychological distress in adults living in all districts of the Kashmir Valley. Thus indicating a need for programmes targeted at improving the mental health of the general population, moving away from an individualistic model of care that is currently the practice in Kashmir. There is a need for intervention trials to establish evidence on mental health programmes that have a positive impact on the mental health of the population. In response to the findings of this study a working group among key mental health stakeholders was established with a commitment to advocating for and implementing programmatic and policy change. It is our hope that there will be a greater commitment to the allocation of necessary resources for the development and trial of mental health interventions in the Kashmir Valley.

## Appendix

Supplementary_File_TableA1.pdf
