# Supplementary material for: Prevalence of anxiety, depression and post-traumatic stress disorder in the Kashmir Valley
Source: BMJ Glob Health. 2017 Oct 15;2(4):e000419. doi: 10.1136/bmjgh-2017-000419 (PMC5654454; doi:10.1136/bmjgh-2017-000419)
Supplement: Supplementary file 1 [file bmjgh-2017-000419supp001.pdf]

Table A1: Sensitivity analysis of weighting for analysis, Kashmir Mental Health Survey 2015

Screening Instrument	Proportion Positive	95% CI		Deff
**PTSD (HTQ)**				
No weighting	22.01%	20.50%	23.60%	1.95
Post stratification weighting†	**19.25%**	**17.45%**	**21.19%**	
**Depression (HSCL)**				
No weighting	46.71%	45.13%	48.30%	1.40
Post stratification weighting†	**41.28%**	**39.20%**	**43.38%**	
**Anxiety (HSCL)**				
No weighting	32.21%	30.67%	33.79%	1.55
Post stratification weighting†	**25.62%**	**23.79%**	**27.54%**	
**Total HSCL-25**				
No weighting	51.44%	49.75%	53.13%	1.59
Post stratification weighting†	**44.79%**	**42.63%**	**46.98%**	

†Post stratification weights were calculated by dividing the proportion of males/females in the census population by the proportion of males/females represented in our sample, this provided a weight of 1.49 for males and 0.73 for females.

Deff = design effect CI = confidence interval, HTQ = Harvard Trauma Questionnaire, HSCL = Hopkins Symptoms Checklist
